# Prophylactic donor-derived CD19 CAR-T cell infusion for preventing relapse in high-risk B-ALL after allogeneic hematopoietic stem cell transplantation

**DOI:** 10.1038/s41375-024-02251-5

**Published:** 2024-04-17

**Authors:** Wenyi Lu, Hairong Lyu, Xia Xiao, Xue Bai, Meng Zhang, Jiaxi Wang, Yedi Pu, Juanxia Meng, Xiaomei Zhang, Haibo Zhu, Ting Yuan, Bing Wang, Xin Jin, Xinping Cao, Zhao Wang, Tianle Xie, Haotian Meng, Alexey V. Stepanov, Alexander G. Gabibov, Yuxin An, Rui Sun, Yu Zhang, Mikhail A. Maschan, Zunmin Zhu, Hongkai Zhang, Mingfeng Zhao

**Affiliations:** 1 Department of Hematology, Institute of Hematology, Henan Provincial People’s Hospital, People’s Hospital of Zhengzhou University, People’s Hospital of Henan University, Zhengzhou, China; 2 Department of Hematology, Tianjin First Central Hospital, Tianjin, China; 3 Department of Hematology, Nankai University Affiliated First Central Hospital, Tianjin, China; 4 Department of Hematology, Beijing Children’s Hospital, Beijing, China; 5 M.M. Shemyakin and Yu.A. Ovchinnikov Institute of Bioorganic Chemistry of the Russian Academy of Sciences, Moscow, Russia; 6 Dmitry Rogachev National Medical Research Center of Pediatric Hematology, Oncology and Immunology, Moscow, Russia; 7 State Key Laboratory of Medicinal Chemical Biology and College of Life Science, Nankai University, Tianjin, China

**Keywords:** Immunotherapy, Bone marrow transplantation, Acute lymphocytic leukaemia

## To the Editor:

Relapse remains a key cause of transplant failure in high-risk B-cell acute lymphoblastic leukemia (B-ALL). Nevertheless, the available maintenance therapy for relapse prevention after allogeneic hematopoietic stem cell transplantation (allo-HSCT) in high-risk B-ALL, especially in Ph-negative B-ALL patients, has limitations and suboptimal efficacy. Notably, donor-derived anti-CD19 chimeric antigen receptor T (CAR-T) cell therapy has emerged as a successful salvage or preemptive strategy in B-ALL patients following allo-HSCT [1–3]. In light of these developments, prophylactic CAR-T cell infusion after allo-HSCT may be a potential strategy to eliminate residual leukemia cells in high-risk B-ALL patients, thereby reducing the risk of relapse prior to the complete immune reconstitution. In this study, we found that prophylactic infusion of CAR-T cells after allo-HSCT in high-risk B-ALL patients resulted in acceptable adverse events and a significantly reduced relapse rate.

Between May 2017 and April 2023, a total of 23 high-risk B-ALL patients received prophylactic donor-derived CAR-T cell infusion after allo-HSCT (ChiCTR 2000041025 and ChiCTR-ONN-16009862). Additionally, a contemporary cohort of 44 high-risk B-ALL patients, who did not receive post-transplant maintenance therapy, was retrospectively identified to serve as a control. The primary endpoint was the safety of CAR-T cell therapy. Secondary outcomes were cumulative incidence of relapse, progression-free survival, and overall survival. Eligibility criteria and methods are detailed in Supplementary Material and Methods.

In the CAR-T group, the median infused cell dose was 2.0 × 10^6^/kg (range: 1.0–4.9 × 10^6^/kg), and the median time from transplantation to CAR-T cell treatment was 122 days (65–315). Among the patients, 16 (69.6%) had a high-risk cytogenetic or molecular profile at diagnosis and seven were Ph+ B-ALL. Seven patients (30.4%) in CAR-T group had detectable minimal residual disease (MRD) or active disease at the time of allo-HSCT, while 11 patients in control group had MRD, which subset of patients are high risk at relapse after allo-HSCT. The main clinical characteristics of the patients were comparable between the CAR-T group and control group (Table 1).

**Table 1:** Patient and transplant characteristics for study group and control group.

Characteristic	CAR-T group (*N* = 23)	Control group (*N* = 44)	*P*
Median age at transplantation, years	32 (9–63)	36 (14–56)	0.953
Sex, *n* (%)			0.120
Male	14 (60.8)	18 (40.9)	
Female	9 (39.1)	26 (59.1)	
High-risk cytogenetic/ molecular risk, *n* (%)	16 (69.6)	24 (54.5)	0.234
Complex cytogenetics, *n* (%)	5 (21.7)	4 (9.1)	0.287
Fusion genes, *n* (%)			
BCR/ABL	7 (30.4)	15 (34.1)	0.762
MLL-rearranged	1 (4.3)	2 (4.5)	1.000
High-risk gene mutations, *n* (%)			
TP53	3 (13.0)	1 (2.3)	0.221
Ph-like	2 (8.7)	2 (4.5)	0.890
IKZF1	1 (4.3)	4 (9.1)	0.832
Extramedullary disease, *n* (%)			0.152
Yes	12 (52.2)	15 (34.1)	
No	11 (47.8)	29 (65.9)	
CAR-T cell therapy before allo-HSCT, *n* (%)			0.917
Yes	17 (73.9)	32 (72.7)	
No	6 (26.1)	12 (27.3)	
Disease status at allo-HSCT, *n* (%)			0.294
CR1	12 (52.2)	23 (52.3)	
CR2	8 (34.8)	20 (45.5)	
CR3	2 (8.7)	1 (2.3)	
Relapse	1 (4.3)	0 (0)	
MRD status before allo-HSCT, *n* (%)			0.935
FCM+	2 (8.7)	4 (9.1)	
FCM+ and PCR+	4 (17.4)	6 (13.6)	
PCR+	1 (4.3)	1 (2.3)	
FCM− and MRD−	16 (69.6)	33 (75.0)	
FCM+ Range before allo-HSCT, *n* (%)			0.132
FCM level (å 1%)	1 (4.3)	0 (0.0)	
FCM level (0.1–1%)	3 (13.0)	2 (4.5)	
FCM level (0.01–0.1%)	2 (8.7)	8 (18.2)	
Donor type, *n* (%)			0.698
Haploidentical	17 (73.9)	35 (79.5)	
Matched related	3 (13.0)	6 (13.6)	
Matched unrelated	3 (13.0)	3 (6.8)	
Karnofsky performance status, *n* (%)			1.000
<80	3 (13.0)	6 (13.6)	
≥80	20 (87.0)	38 (86.4)	
Conditioning regimen, *n* (%)			0.996
Bu/Cy	7 (30.4)	13 (29.5)	
TBI/Cy	14 (60.9)	27 (61.4)	
Others	2 (8.7)	4 (9.1)	
CD34^+^ cells, ×10^6^/kg	4.6 (1.4–8.5)	4.6 (2.3–9.6)	0.875
MNC, ×10^8^/kg	6.1 (3.2–10.0)	5.8 (2.9–11.3)	0.850
Time of leukocyte engraftment, day	12 (10–29)	12 (10–30)	0.238
Time of platelet engraftment, day	16 (10–31)	15 (8–62)	0.974
Follow-up time, months	24.9 (6.7–67.7)	23.0 (4.4–61.8)	

*Bu/Cy* busulfan/cyclophosphamide-based conditioning regimen, *MNC* mononuclear cells, *TBI/Cy* total body irradiation/cyclophosphamide-based conditioning regimen, *MRD* minimal residual disease, *FCM* flow cytometry.

Toxicities associated with CAR-T cell therapy are summarized in Supplementary Table S1. Cytokine release syndrome (CRS) was the predominant non-hematological toxicity. Our data indicate that 47.8% of patients experienced grade 1/2 CRS, with no cases of grade 3–4 CRS. The median time to onset of CRS was 1 day (0–6), and the median duration was 2 days (1–10). In response to CRS, three patients required an escalated steroid regimen, and two received tocilizumab. None of the patients required intensive care unit admission for the management of CRS. Furthermore, there were no cases of ICANS at any grade following the CAR-T cell treatment.

Hematopoietic toxicity emerged as the most prevalent and severe adverse event, with 95.7% of the patients experiencing at least monolineage cytopenia within 28 days after CAR-T cell infusion. The median onset time of cytopenia was 1.5 days (range: 1–9). Grade 3–4 leukopenia, thrombocytopenia, and anemia were observed in 65.2%, 43.5%, and 21.7% of the patients, respectively, which are consistent with those reported in other trials involving CD19 CAR-T cell therapy (Supplementary Table S1) [4, 5]. Importantly, the majority of (13/22) hematopoietic toxicities were reversed by hematopoietic growth factor and supportive therapy. However, a subset of nine patients exhibited persistent cytopenia post CAR-T cell therapy, all of whom demonstrated marked improvement of cytopenia upon the escalation of prednisone dosage. Following this intervention, leukocyte counts were restored to normal levels in 66.7% (6/9) of these patients, while platelet and hemoglobin levels were similarly normalized in 55.6% (5/9) of the cases. The median recovery times for leukopenia, anemia, and thrombocytopenia were 12 days (range: 2–81 days), 11 days (range: 2–98 days), 22 days (range: 6–147 days), respectively, to either complete recovery or improvement to Grade 2 severity.

In addition to the routine complication, acute graft-versus-host disease (aGVHD) is also a major concern in allogeneic CAR-T cell therapy. Several studies have reported that donor-derived CD19 CAR-T cells elicit a low occurrence of aGVHD [6–9]. In our study, three patients developed aGVHD after CAR-T cell infusion: two with grade 2 and one with grade 3. The median time of onset was 20 days (range: 7–25 days) after CAR-T cell infusion. Curiously, two of the three aGVHD patients had discontinued basal prednisolone before CAR-T cell infusion, which may contribute to the occurrence of aGVHD. Infection is another critical concern, with three patients succumbing to various infections (fungal, sepsis, and COVID-19). However, the 2-year non-relapse mortality was comparable between the CAR-T and control groups [10.3% (95% CI: 0–24%) vs. 14.4% (95% CI: 3.7–25.1%), *P* = 0.847, Supplementary Fig. S1A].

**Fig. 1:**
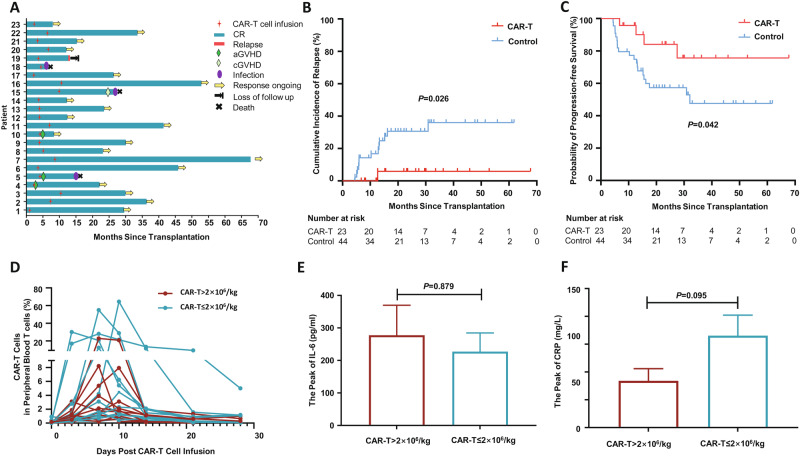
Clinical outcomes, CAR-T proliferation, and peripheral blood biomarkers after donor-derived CD19 CAR-T cell infusion. **A** Swimmer plot depicting clinical outcomes after CD19 CAR-T cell maintenance therapy. **B** The 2-year cumulative incidence of relapse after CD19 CAR-T cell infusion. **C** The probability of 2-year progression-free survival after CD19 CAR-T cell infusion. **D** Proliferation of CAR-T cells in patients who received a high dose (>2 × 10^6^/kg, *n* = 10) and those who received a low dose (≤2 × 10^6^/kg, *n* = 13) of CAR-T cells. **E** The peak level of serum IL-6 after CAR-T cell infusion in high dose group (*n* = 10) or low dose group (*n* = 13). **F** The peak level of serum CRP (c-reactive protein) after CAR-T cell infusion in high dose group (*n* = 10) or low dose group (*n* = 13). Data are represented as mean ± SEM.

At the last follow-up, only one patient in the CAR-T group had an extramedullary relapse, resulting in a significantly lower 2-year cumulative incidence of relapse of 5.6% (95% CI: 0.0–15.3%) compared to the control group (28.8%, 95% CI: 15.1–42.5%, *P* = 0.026, Fig. 1A, B). Significantly, at the time of this relapse, both the patient’s bone marrow and cerebrospinal fluid were found to be in remission. This case highlights the need for further research into the causes of extramedullary relapse after CAR-T therapy, despite bone marrow and CNS remission. Furthermore, the 2-year progression-free survival rate was higher in the CAR-T group [84.0% (95% CI: 67.1–100.0%) vs. 57.3% (95% CI: 42.2–72.4%), *P* = 0.042, Fig. 1C]. Nevertheless, the 2-year overall survival rate was not statistically different between the two groups [89.3% (95% CI: 75.0–100.0%) vs. 75.4% (95% CI: 62.1–88.7%), *P* = 0.324, Supplementary Fig. S1B]. This may be attributed to the small sample size of the study. Additionally, the implementation of advanced salvage treatments by the majority of patients who relapsed—such as salvage CAR-T cell therapy, anti-CD22 antibody, and anti-CD38 antibody—could be another factor. The transition to these innovative salvage approaches might necessitate a longer follow-up period to fully discern the impact of prophylactic CAR-T cell therapy on overall survival.

In our study, we chose prophylactic CAR-T cell therapy for seven Ph+ ALL patients over conventional tyrosine kinase inhibitor (TKI) treatment due to TKI resistance or adverse reactions in two patients, and the proven efficacy of CD19 CAR-T cells in eradicating refractory and residual leukemia cells [10, 11]. Patients were rigorously monitored for MRD, CAR-T persistence, and B-cell aplasia, with supplemental TKI therapy recommended if CAR-T and B-cell aplasia resolved, to lower relapse risk. This approach, provided at no cost, aimed to alleviate the financial burden of TKI maintenance, showcasing a multifaceted, patient-centered treatment strategy. Notably, all seven Ph+ B-ALL patients who received post-transplant CAR-T cell maintenance therapy alone remained in durable remission, indicating CAR-T as a possible alternative to TKI post-transplant in Ph+ ALL.

We further monitored the kinetics of CAR-T cells and associated biomarkers in patients. As illustrated in Fig. 1D, the median peak of CAR-T cell expansion occurred at day 7 (range: 3–14) and most were undetectable by day 28. Importantly, we did not observe a difference in the proliferation and persistence of CAR-T cells between infusions characterized by doses greater than and those less than or equal to 2 × 10^6^/kg. (Fig. 1D). Furthermore, the peak levels of IL-6 and c-reactive protein in serum were not apparently different, suggesting an absence of correlation between the infused cell dose and the proliferation of CAR-T cells, as well as the cytokine response (Fig. 1E, F). Oink proteomics analysis further revealed a significant upregulation in serum protein levels of SIT1, CD28, and IL2RB after CAR-T cell infusion, and KEGG analysis showed that differentially expressed proteins were associated with JAK-STAT and PI3K-AKT pathways (Supplementary Fig. S2). These data suggest that prophylactic infusion of donor-derived CAR T cells after transplantation may also activate important immune signaling pathways. Noteworthy observations also included B-cell aplasia following CAR-T cell infusion, with a median duration of 233 days (range: 39–995 days), which may indirectly reflect the efficacy of CD19 CAR-T cells (Supplementary Fig. S3).

Despite the potential benefits, the present study has some limitations. First, the sample size was relatively small. Moreover, the presence of patient heterogeneity adds complexity to the interpretation of the results. Another limitation of this investigation pertains to the retrospective nature of the analysis, which resulted in variability in both the timing of CAR-T cell administration and the dosing regimen used for maintenance therapy. However, by comparing with a contemporary control, the efficacy for donor-derived CAR-T cell maintenance was preliminarily estimated.

In conclusion, our study suggests that prophylactic CAR-T cell therapy infusion may be a feasible strategy to achieve durable remission in high-risk B-ALL patients after transplantation. Furthermore, the toxicities associated with this treatment were acceptable. Given the relatively small sample size and non-randomized study design, future larger-scale prospective randomized multicenter trials are needed to provide more robust evidence to support the use of prophylactic CAR-T cell therapy in high-risk B-ALL patients, especially those patients who are MRD positive before or after allo-HSCT.

### Supplementary information

Supplementary materials

## Data Availability

All data generated and analyzed in this study are available in the published article and its additional files. For all original data from this study, please contact the corresponding author via mingfengzhao@sina.com. All shared data will be de-identified and made available upon reasonable request.

## References

[1] Liu J, Zhong JF, Zhang X, Zhang C (2017). Allogeneic CD19-CAR-T cell infusion after allogeneic hematopoietic stem cell transplantation in B cell malignancies. J Hematol Oncol.

[2] Hua JS, Zhang J, Zhang XY, Wu XX, Zhou LL, Bao XB (2021). Donor-derived anti-CD19 CAR T cells compared with donor lymphocyte infusion for recurrent B-ALL after allogeneic hematopoietic stem cell transplantation. Bone Marrow Transplant.

[3] Zhao XY, Xu ZL, Mo XD, Chen YH, Lv M, Cheng YF (2022). Preemptive donor-derived anti-CD19 CAR T-cell infusion showed a promising anti-leukemia effect against relapse in MRD-positive B-ALL after allogeneic hematopoietic stem cell transplantation. Leukemia.

[4] Si X, Gu T, Liu L, Huang Y, Han Y, Qian P (2022). Hematologic cytopenia post CAR T cell therapy: etiology, potential mechanisms and perspective. Cancer Lett.

[5] Jain T, Knezevic A, Pennisi M, Chen Y, Ruiz JD, Purdon TJ (2020). Hematopoietic recovery in patients receiving chimeric antigen receptor T-cell therapy for hematologic malignancies. Blood Adv.

[6] Zhang C, Wang XQ, Zhang RL, Liu F, Wang Y, Yan ZL (2021). Donor-derived CD19 CAR-T cell therapy of relapse of CD19-positive B-ALL post allotransplant. Leukemia.

[7] Kochenderfer JN, Dudley ME, Carpenter RO, Kassim SH, Rose JJ, Telford WG (2013). Donor-derived CD19-targeted T cells cause regression of malignancy persisting after allogeneic hematopoietic stem cell transplantation. Blood.

[8] Brudno JN, Somerville RP, Shi V, Rose JJ, Halverson DC, Fowler DH (2016). Allogeneic T cells that express an anti-CD19 chimeric antigen receptor induce remissions of B-cell malignancies that progress after allogeneic hematopoietic stem-cell transplantation without causing graft-versus-host disease. J Clin Oncol.

[9] Ghosh A, Smith M, James SE, Davila ML, Velardi E, Argyropoulos KV (2017). Donor CD19 CAR T cells exert potent graft-versus-lymphoma activity with diminished graft-versus-host activity. Nat Med.

[10] Lee DW, Kochenderfer JN, Stetler-Stevenson M, Cui YK, Delbrook C, Feldman SA (2015). T cells expressing CD19 chimeric antigen receptors for acute lymphoblastic leukaemia in children and young adults: a phase 1 dose-escalation trial. Lancet.

[11] Lu W, Wei Y, Cao Y, Xiao X, Li Q, Lyu H (2021). CD19 CAR-T cell treatment conferred sustained remission in B-ALL patients with minimal residual disease. Cancer Immunol Immunother.

