# Supplementary material for: Prophylactic donor-derived CD19 CAR-T cell infusion for preventing relapse in high-risk B-ALL after allogeneic hematopoietic stem cell transplantation
Source: Leukemia. 2024 Apr 17;38(6):1419–22. doi: 10.1038/s41375-024-02251-5 (PMC11147756; doi:10.1038/s41375-024-02251-5)
Supplement: Supplementary file 1 — Supplementary materials [file 41375_2024_2251_MOESM1_ESM.docx]

**Supplementary materials**

**Material and methods**

**Study Plan**

In this study, we included a total of 23 B-ALL patients at high risk of relapse after allo-HSCT who received prophylactic donor-derived CAR-T cells therapy between May 2017 and April 2023 (ChiCTR 2000041025 and ChiCTR-ONN-16009862). This study involved individuals aged 1 to 70 years, with Eastern Cooperative Oncology Group (ECOG) scores ranging from 0 to 3, who met the eligibility criteria for CAR-T cell therapy. B-ALL at high risk of relapse after transplantation was defined based on at least one of the following criteria: high white blood cell count (≥30×10^9^/L) at diagnosis, failure of primary induction requiring more than two cycles of therapy, refractory or non-remission at transplantation at the time of allo-HSCT, beyond CR1 at the time of allo-HSCT, and high-risk cytogenetic or molecular abnormalities such as t (9;22) or BCR-ABL1 positive ALL, Ph-like ALL, KMT2A rearrangements, complex karyotype (≥5 unrelated clonal abnormalities) or hypodiploid cytogenetics at diagnosis. Patients were excluded from this study if they had MRD or relapsed disease prior to CAR-T cell therapy.

To serve as a control, a contemporary cohort of 44 high-risk B-ALL patients who did not receive prophylactic therapy after transplantation were enrolled. To ensure that the two groups are comparable, the patients who failed to achieve MRD- CR post-transplantation were also excluded from the control group. This study was approved by the Ethics Committee of Tianjin First Central Hospital, and it was conducted in accordance with the Good Clinical Practice Guidelines of the International Conference on Harmonization. Informed consent was obtained from all patients after thorough discussion of the potential risks and adverse effects of the therapy. The primary outcome was the safety of CAR-T therapy. Secondary outcomes were cumulative incidence of relapse, progression-free survival, and overall survival. The cut-off date for follow-up was Nov 11th, 2023. Considering the relatively small population size receiving CAR-T cell therapy post-allogeneic hematopoietic stem cell transplantation, we established our sample size based on references to similar studies [1,2].

**Transplantation procedure**

Conditioning regimens for myeloablative transplantation were administered according to established protocols. For matched sibling transplants, the conditioning regimen consisted of either total body irradiation with a dose range of 8-10Gy or busulfan at 8mg/kg, along with cyclophosphamide (40mg/kg) intravenously on days -6 and -5, fludarabine (30 mg/m^2^) on days -4 to -2, and cytarabine (2g/m^2^/day) on days -4 to -2.

For haploidentical and unrelated donor HSCT, total body irradiation (8-10Gy) or busulfan (8mg/kg) was administered on days -9 to -7, followed by anti-thymocyte globulin at a dose of 7.5-15mg/kg from day -7 to day -4, cyclophosphamide (40mg/kg) on days -6 and -5, fludarabine(30mg/m^2^) and cytarabine (4g/m^2^) on days -4 to -2. For patients received haploidentical donor HSCT, 4-5/6 HLA loci matched cord blood was infused on the day after haploidentical stem cells infusion. All transplant recipients received graft-versus-host-disease (GVHD) prophylaxis with cyclosporine A, mycophenolate mofetil, and a low-dose, short-course methotrexate. Supportive care was provided as previously described [3].

**MRD monitoring**

Evaluation of bone marrow samples was systematically conducted prior to the initiation of each chemotherapy cycle and prior to the transplantation. Following transplantation, MRD was performed on the 14th, 28th day and then every month thereafter. MRD was monitored using flow cytometry (FCM, sensitivity 1/10,000) and real-time quantitative polymerase chain reaction (RQ-PCR) /deep sequencing (sensitivity 1/10,000). For the detection of MRD via FCM, an panel of antibodies targeting CD10, CD19, CD20, CD22, CD34, CD38, CD45, cTdT, and CD79a was utilized. The FCM analyses was performed with the CANTO II flow cytometer (BD Biosciences). Real-Time Quantitative Polymerase Chain Reaction (RQ-PCR) or deep sequencing was also performed to evaluate MRD, according to the expression of BCR/ABL, MLL, TP53, or IKZF1 etc.

**Preparation of CAR-T cells**

Specifically, collection of peripheral blood mononuclear cells from the donor was conducted approximately three months following transplantation, provided there was no severe GVHD, infections, or any other contraindications for CAR-T cell therapy. Donor-derived CD19 CAR-T cells were generated as detailed below. Briefly, CD3^+^ T lymphocytes were isolated from peripheral blood mononuclear cells and activated with CD3/CD28 beads and IL-2. The T lymphocytes were then transduced with a lentiviral vector containing the anti-CD19 single-chain fragment variable CAR gene and the CD28 costimulatory domain, followed by expansion for a further 10-12 days.

**Infusion of CAR-T cells**

The majority of patients enrolled in this study were being administered low-dose steroids and/or immunosuppressants to reduce the onset of GVHD at the time of CAR-T cell treatment. Importantly, these treatments did not need to be discontinued prior to CAR-T cell infusion. Prior to infusion, patients received Cy (250mg/m^2^) and fludarabine (25mg/m^2^) for a period of one to three days, depending on their complete blood count and bone marrow assessment. Additionally, patients diagnosed with Ph^+^ ALL who received CAR-T cell therapy were not eligible to receive post-transplant TKI maintenance therapy, unlike the control group.

**Efficacy evaluations**

Bone marrow evaluations were performed on the 14th and 28th day after the initial CAR-T cell infusion, and every two months thereafter. The presence of MRD in bone marrow samples were monitored by flow cytometry, real-time quantitative polymerase chain reaction (PCR), and deep sequencing analysis.

**Safety evaluations**

The Common Terminology Criteria for Adverse Events version 5 was used to assess adverse events. Cytokine release syndrome and immune effector cell-associated neurotoxicity syndrome were graded according to the American Society for Transplantation and Cellular Therapy consensus grading system [4]. Acute GVHD was staged and graded based on the criteria published by Przepiorka et al [5].

**Olink Proteomic Measurement**

The proximity extension assay was used to measure 92 proteins before and seven days after CAR-T cell infusion, using the Olink platform. During the incubation phase, 92 antibody pairs that were labelled with DNA oligonucleotides were allowed to bind to their respective protein samples for 16-22 hours. In the extension and amplification phase, the proximity oligonucleotides hybridized and were extended using a DNA polymerase, resulting in a DNA barcode that was amplified by PCR. The amount of each DNA barcode was then quantified using microfluidic qPCR and quantified using Olink® Signature Q100. Finally, the data was read using Olink® NPX Signature software.

**Statistical analysis**

The Mann-Whitney U test or Student’s t-test was used to compare patient characteristics between different groups. The variance across the groups was assessed for homogeneity. When variances were found to be equivalent, a t-test was applied to determine statistical significance; conversely, in situations where variances were not equal, the Mann-Whitney U test was utilized. The Kaplan-Meier method was used to generate overall survival and progression-free survival curves, which were then compared using the log-rank test. Competing risk analysis was used to estimate cumulative incidence of relapse, and Gray's test was used to compare CIR curves. Statistical analyses were performed using SPSS version 25.0, GraphPad Prism version 7.0 and R software version 4.1.3. Statistical significance was defined as *P*< 0.05, two-sided.

**Table S1. The adverse effect after CAR-T cell therapy**

| **Adverse event, NO. (%)** | **Total (N=23)** | | |  |
| --- | --- | --- | --- | --- |
|  | **Grade1** | **Grade 2** | **Grade 3** | **Grade 4** |
| **Inflammation-related event** |  |  |  |  |
| Fever | 9 (39.1) | 2 (8.7) | 0 (0) | 0 (0) |
| Febrile neutropenia | 0 (0) | 0 (0) | 10 (43.5) | 0 (0) |
| Cytokine release syndrome | 9 (39.1) | 2 (8.7) | 0 (0) | 0 (0) |
| **Haematological event** |  |  |  |  |
| Anemia | 1 (4.3) | 9 (39.1) | 5 (21.7) | 0 (0) |
| Leukopenia | 0 (0) | 6 (26.1) | 8 (34.8) | 7 (30.4) |
| Neutropenia | 1 (4.3) | 5 (21.7) | 7 (30.4) | 6 (26.1) |
| Lymphopenia | 0 (0) | 6 (26.1) | 7 (30.4) | 7 (30.4) |
| Thrombocytopenia | 2 (8.7) | 4 (17.4) | 5 (21.7) | 5 (21.7) |
| **Nervous system event** | 0 (0) | 0 (0) | 0 (0) | 0 (0) |
| **Chemical laboratory abnormalities** |  |  |  |  |
| Alanine aminotransferase increased | 4 (17.4) | 1 (4.3) | 0 (0) | 0 (0) |
| Aspartate aminotransferase increased | 4 (17.4) | 1 (4.3) | 0 (0) | 0 (0) |
| Gamma glutamyl transpeptidase increased | 5 (21.7) | 3 (13.0) | 2 (8.7) | 0 (0) |
| Blood bilirubin increased | 2 (8.7) | 0 (0) | 0 (0) | 0 (0) |
| Serum creatinine increased | 0 (0) | 0 (0) | 0 (0) | 0 (0) |
| Fibrinogen decreased | 1 (4.3) | 0 (0) | 0 (0) | 0 (0) |

**Figure S1**


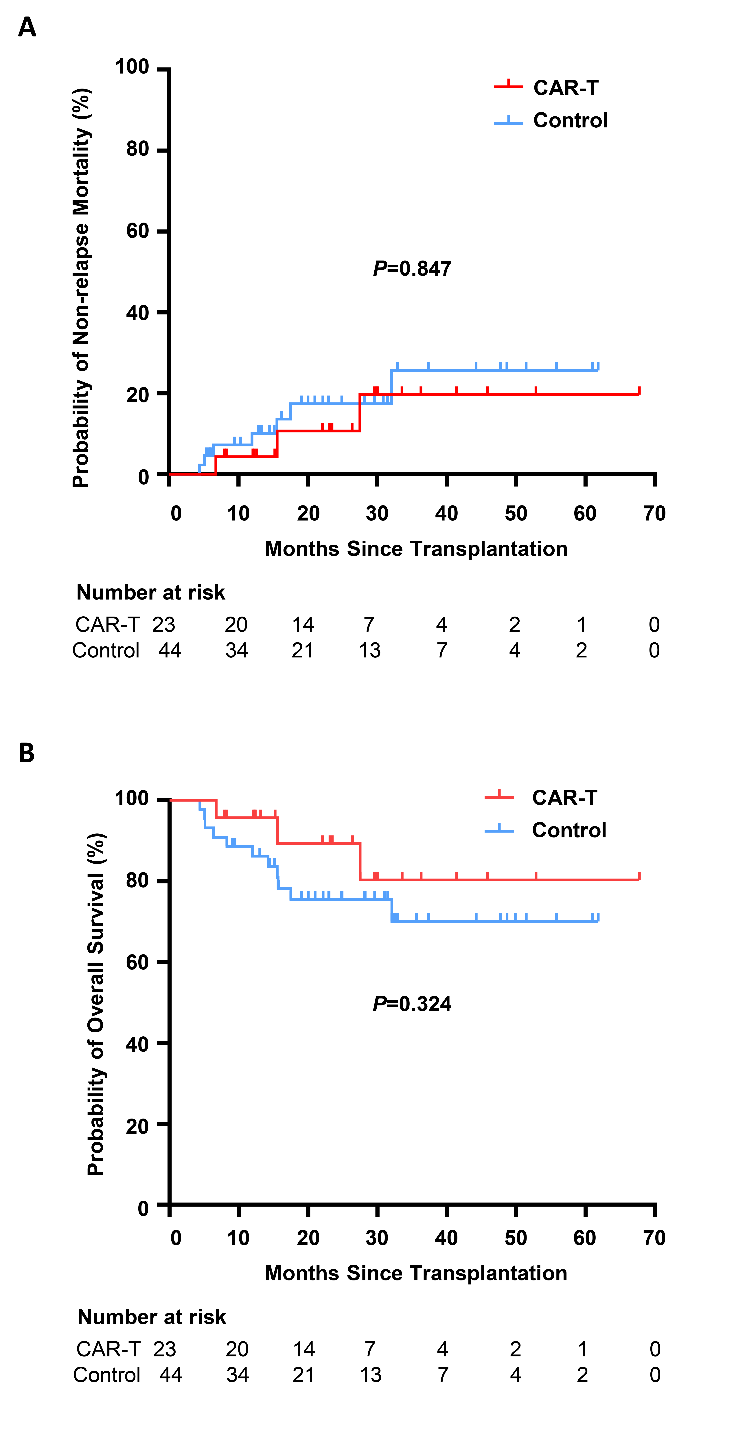


**Figure S1. The outcomes after donor-derived CD19 CAR-T cell infusion.** (A) The probability of two-year non-relapse mortality after CD19 CAR-T cell infusion. (B) The probability of two-year overall survival after CD19 CAR-T cell infusion.

**Figure S2**

**
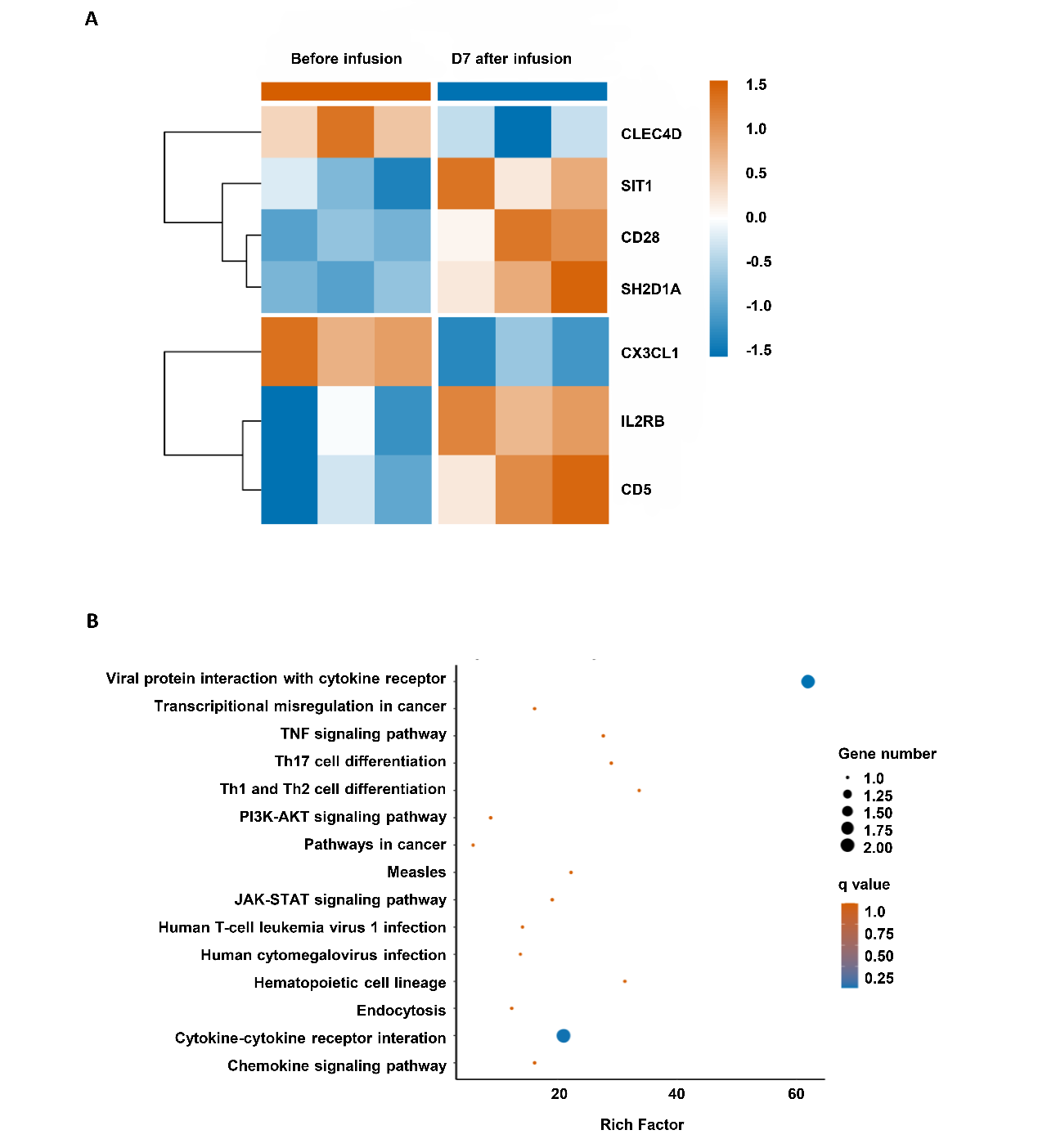
**

**Figure S2. Serum proteomic profiling in three patients before and 7 days after CAR-T cell infusion.** (A) The heatmap of protein differential expression before and 7 days after CAR-T cell infusion. (B) KEGG Pathway enrichment of significant differential expression proteins.

**Figure S3**


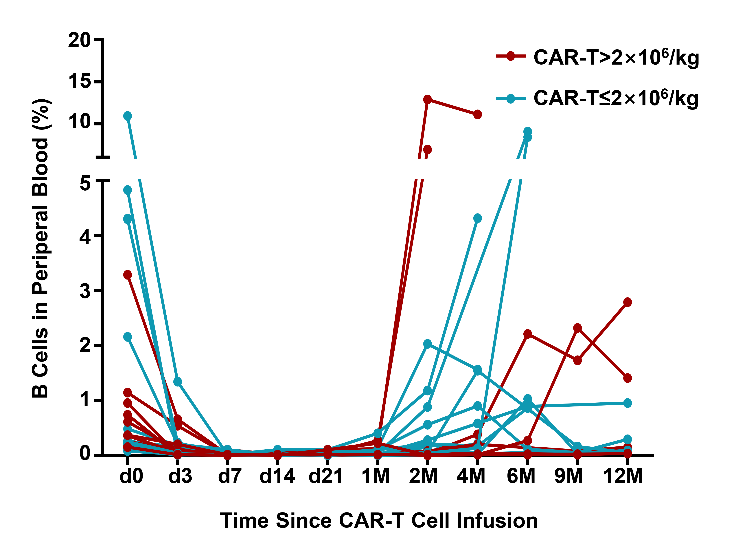


**Figure S3. Peripheral blood B-cell kinetics in patients receiving high or low doses of CAR-T cells**

**References:**

1. Zhao XY, Xu ZL, Mo XD, Chen YH, Lv M, Cheng YF, et al. Preemptive donor-derived anti-CD19 CAR T-cell infusion showed a promising anti-leukemia effect against relapse in MRD-positive B-ALL after allogeneic hematopoietic stem cell transplantation. Leukemia. 2022 ;36(1):267-270.
2. Gaballa MR, Banerjee P, Milton DR, Jiang X, Ganesh C, Khazal S, et al. Blinatumomab maintenance after allogeneic hematopoietic cell transplantation for B-lineage acute lymphoblastic leukemia. Blood. 2022;139(12):1908-1919.
3. Lu W, Jin X, Lyu H, Bai X, Zhu H, Li X, et al. A Prospective Trial Comparing Haploidentical Donor Transplantation With Cord Blood Versus HLA-Matched Sibling Donor Transplantation for Hematologic Malignancy Patients. Cell Transplant. 2022; 31:9636897221076050.
4. Lee DW, Santomasso BD, Locke FL, Ghobadi A, Turtle CJ, Brudno JN, et al. ASTCT Consensus Grading for Cytokine Release Syndrome and Neurologic Toxicity Associated with Immune Effector Cells. Biol Blood Marrow Transplant. 2019;25(4):625-38.
5. Przepiorka D, Weisdorf D, Martin P, Klingemann HG, Beatty P, Hows J, et al. 1994 Consensus Conference on Acute GVHD Grading. Bone Marrow Transplant. 1995;15(6):825-28.
